# Logistic Regression Model Using Scheimpflug-Placido Cornea Topographer Parameters to Diagnose Keratoconus

**DOI:** 10.1155/2021/5528927

**Published:** 2021-05-18

**Authors:** Emre Altinkurt, Ozkan Avci, Orkun Muftuoglu, Adem Ugurlu, Zafer Cebeci, Kemal Turgay Ozbilen

**Affiliations:** ^1^Istanbul University, Istanbul Faculty of Medicine, Department of Ophthalmology, Fatih/Capa, Istanbul 34093, Turkey; ^2^FEBO, Professor of Ophthalmology, Koc University, Faculty of Medicine Department of Ophthalmology, Zeytinburnu/İstanbul 34010, Turkey; ^3^Erzincan University, Faculty of Medicine, Department of Ophthalmology, Fatih, Erzincan 24100, Turkey

## Abstract

**Purpose:**

Diagnose keratoconus by establishing an effective logistic regression model from the data obtained with a Scheimpflug-Placido cornea topographer.

**Methods:**

Topographical parameters of 125 eyes of 70 patients diagnosed with keratoconus by clinical or topographical findings were compared with 120 eyes of 63 patients who were defined as keratorefractive surgery candidates. The receiver operating character (ROC) curve analysis was performed to determine the diagnostic ability of the topographic parameters. The data set of parameters with an AUROC (area under the ROC curve) value greater than 0.9 was analyzed with logistic regression analysis (LRA) to determine the most predictive model that could diagnose keratoconus. A logit formula of the model was built, and the logit values of every eye in the study were calculated according to this formula. Then, an ROC analysis of the logit values was done.

**Results:**

Baiocchi Calossi Versaci front index (BCV_f_) had the highest AUROC value (0.976) in the study. The LRA model, which had the highest prediction ability, had 97.5% accuracy, 96.8% sensitivity, and 99.2% specificity. The most significant parameters were found to be BCV_f_ (*p*=0.001), BCV_b_ (Baiocchi Calossi Versaci back) (*p*=0.002), posterior rf (apical radius of the flattest meridian of the aspherotoric surface in 4.5 mm diameter of the cornea) (*p*=0.005), central corneal thickness (*p*=0.072), and minimum corneal thickness (*p*=0.494).

**Conclusions:**

The LRA model can distinguish keratoconus corneas from normal ones with high accuracy without the need for complex computer algorithms.

## 1. Introduction

Keratoconus is an ectatic disorder that is characterized by progressive stromal thinning and protrusion of the cornea with irregular astigmatism [1]. Ectasia progression after refractive surgery in patients with keratoconus has been reported in previous studies [2]. The prevalence of keratoconus is higher among refractive surgery candidates compared to the general population [3], and operating on a cornea with keratoconus can cause corneal ectasia after refractive surgery [4]. Topography systems are very useful in the diagnosis of keratoconus. Still, an exact diagnosis is difficult because threshold criteria remain to be defined. Moreover, examining each parameter in the topography device one by one takes time. This study aimed to gauge the most useful parameters of the Sirius Scheimpflug-Placido topographer in determining keratoconus eyes from normal eyes and to find an accurate logistic regression model for diagnosing keratoconus with these parameters.

## 2. Patients and Methods

The study was conducted in agreement with the ethical standards established in the Declaration of Helsinki and endorsed by the local clinical research ethics committee. Informed consent was obtained from all patients. The study consisted of 120 eyes from 63 patients who had undergone keratorefractive surgery (normal group) and 125 eyes from 70 patients who were diagnosed with keratoconus (keratoconus group) between November 2012 and May 2015. The parameters provided by the Scheimpflug-Placido topographer (Sirius 3 D Rotating Scheimpflug Camera and Topography System, Costruzione Strumenti Oftalmici, Italy) were evaluated retrospectively. Excluded were patients who were pregnant or lactating and who had corneal scarring, dry eye, concomitant corneal or ocular disease, previous corneal collagen crosslinking, ocular surgery, or trauma. Subjects were divided into normal and keratoconus groups.

All patients in the keratoconus group were diagnosed as keratoconus on the basis of topographic signs. In this study, keratoconus is defined as any eye that has an abnormal localized steepening or an asymmetric bow tie pattern with or without skewed axes, combined with at least one of the following signs: central corneal thickness <500 *µ*m, oblique cylinder >1.5 diopter (D), steep keratometry >47 D, or clinical keratoconus in the fellow eye [5, 6]. Myopic laser refractive surgery patients who were assigned to the normal group had preoperative sphere < −6.50 D and cylinder < −3.00 D without an irregular corneal topographic pattern. No forme fruste keratoconus patients were included among refractive surgery candidates.

Corneal scans were done under scotopic conditions without pupillary dilation by the same experienced operator using the Sirius Scheimpflug-Placido topographer. Acceptable-quality maps with at least 9.0 mm of corneal coverage were used in the study. All wavefront aberrations were performed for a 6 mm diameter pupil. The following parameters were recorded and analyzed: simulated keratometry measurements consisting of flat (Sim-K1) and steep (Sim-K2) corneal power; corneal dioptric power in the flattest and steepest meridian for both corneal surfaces in the 3.0 mm central corneal zone (anterior Ø = 3 mm K1, anterior Ø = 3 mm K2, posterior Ø = 3 mm K1, posterior Ø = 3 mm K2) and 5.0 mm central corneal zone (anterior Ø = 5 mm K1, anterior Ø = 5 mm K2, posterior Ø = 5 mm K1, posterior Ø = 5 mm K2); minimum corneal thickness (TCT), keratometry of the steepest point (K_max_) on the anterior tangential map, central corneal thickness (CCT), distance from the corneal endothelium to the anterior surface of the lens (AD), distance of the thinnest point to the center of the cornea (TCTr), distance of Kmax to the center of the cornea (Apex *r*), and corneal volume in the 10 mm central corneal zone (CV); symmetry index front (SI_f_) and back (SI_b_); Baiocchi Calossi Versaci index front (BCV_f_) and back (BCV_b_), keratoconus vertex index front (KV_f_) and back (KV_b_); apical radius of the flattest (anterior rf, posterior rf) and steepest meridian (anterior rs, posterior rs) of the aspherotoric surface in the 4.5 mm zone of the cornea front and back, mean asphericity in the 4.5 mm zone of the cornea front (anterior Q) and back (posterior Q), and root mean square values per unit area in the 4.5 mm zone of the cornea front (anterior RMS/A) and back (posterior RMS/A); total wavefront error (OPD), higher-order aberrations (HOAs), astigmatism Z (2, ±2), coma aberration Z (3, ±1), spherical aberration Z (4, 0), and residual HOAs (noncoma, nonspherical) for total, anterior, and posterior surfaces of cornea; and Kmax/TCT and Kmax^2^/TCT values.

All data were analyzed with the Statistical Package for the Social Sciences (SPSS) software (version 21.0, SPSS, Inc.). The Kolmogorov–Smirnov method was used to test the variables for normal distribution. Categorical variables were analyzed using the chi-square test. In the analysis of the difference between the two groups regarding quantitative variables, the normally distributed variables were tested using independent samples *t*-test, and the not normally distributed variables were tested with the Mann–Whitney *U* test. Receiver operating characteristic (ROC) curves were used to evaluate the accuracy of the parameters. The area under the curve (AUROC), sensitivity, specificity, and cut-off values that matched the maximum AUROC were calculated and compared between the two groups. [7] The diagnostic power of the parameters in the study was defined according to the AUROC values as excellent (0.90–1.00), good (0.80–0.89), fair (0.70–0.79), poor (0.60–0.69), and very poor (0.50–0.59). [8] The data set of the parameters with AUROC values greater than 0.9 was analyzed with logistic regression analysis (LRA) to determine the simplest model that could diagnose keratoconus.

The predictive capability of the eight models was investigated using LRA: Model 1 was a cluster of anterior Ø = 3 mm K2, posterior Ø = 3 mm K2, K_max_, CCT, and TCT. Model 2 was a cluster of anterior shape parameters (anterior RMS/A, anterior rs, anterior rf, and anterior Q). Model 3 was a cluster of posterior shape parameters (posterior RMS/A, posterior rs, posterior rf, and posterior Q). Model 4 was an addition of Model 2 and Model 3. Model 5 consisted of anterior and posterior keratoconus indices (BCV_f_, BCV_b_, KV_f_, KV_b_, SI_f_, and SI_b_). Model 6 was a cluster of all corneal aberrations.

The significant parameters in these models were selected and gathered in different combinations with the other parameters in the study to find Model 7 and Model 8, the most predictive models. The logit formula of Model 8 was built. Then, the logit value of every eye in the study was calculated according to the logit formula of Model 8. ROC analysis of the logit values was performed to find the cut-off value of the logit formula. The results were evaluated according to a confidence interval of 95%, and a *p* value <0.05 was considered statistically significant.

## 3. Results

There was no statistically significant difference between the keratoconus (mean age 31.03 ± 9.62 years) and normal groups regarding age (mean age 31.03 ± 8.05 years) distribution (*p* > 0.05). All topographic parameters of the two groups were statistically significantly different (Tables 1 and 2). The ROC curve analysis of the topographic parameters with AUROC values less than 0.9 is in Table 3 and that with AUROC values greater than 0.9 is in Table 4. The AUROC values of the anterior aberrations were greater than the AUROC values of total aberrations; the AUROC values of the anterior keratoconus indices were greater than the AUROC values of the posterior keratoconus indices, and the AUROC values of the keratoconus indices were greater than the AUROC values of the summary indices and K readings. BCV_f_ had the highest AUROC value (0.976) in the study.

The LRA models built for differentiating keratoconus eyes from normal eyes are in Table 5. The most successful LRA was defined in Model 8 with an accuracy of 97.5%, sensitivity of 96.8%, and specificity of 99.2% (−2 log-likelihood score: 42.461). It is formulated as follows: logit = 3,471 + (5,431x BCV_f_) + (6,533x BCV_b_) + (−1.883x posterior rf) + (−0,031x CCT) + (−0,007x TCT). In Model 8, the significance levels were BCV_f_ (*p*=0.001), BCV_b_ (*p*=0.002), posterior rf (*p*=0.005), CCT (*p*=0.072), and TCT (*p*=0.494). The AUROC and cut-off values of the logistic function formula of Model 8 were 0.992 and −0,0225, respectively.

## 4. Discussion

Many technologies have been developed to distinguish keratoconus eyes from normal eyes. The efficacy of Scheimpflug-Placido imaging in differentiating keratoconus eyes from normal eyes has been investigated in previous studies [9].

LRA was used to determine the most predictive model for keratoconus detection in this study, and our LRA model (model 8) can differentiate keratoconus eyes from normal eyes with high accuracy (97.5%), sensitivity (96.8%), and specificity (99.2%). Ucakhan et al. [10] reported a similar performance with their logistic regression model (sensitivity 97.7% and specificity 95.2%) with 11 topographic parameters, while our LRA model can perform similarly with only five parameters. We believe that using five parameters will save time for ophthalmologists. The logistic function formula in our study can be used with an Excel file (Microsoft Office, Microsoft Corp., Redmond, WA, USA). Results greater than the cut-off value (−0, 1512) will indicate keratoconus diagnosis.

There are keratoconus detection algorithms and computer-aided diagnosis systems based on the data of Scheimpflug corneal topography devices in the literature. These have a discriminating ability that is higher than our regression model with an accuracy of 97.2%–99.5% [11–14] and sensitivity of 99.1%–100%. [11, 15], but the LRA model in our study can diagnose keratoconus without the need for complex computer algorithms and software.

The BCV_f_ was found to be the most significant parameter in the logistic regression model (*p*=0.001). It is obtained by a linear combination of vertical trefoil Z (3, −3), vertical coma Z (3, −1), horizontal coma Z (3, +1), primary spherical aberration Z (4, 0), and second-order vertical coma Z (5, −1) and by processing them with a function of the coma axis on the anterior Zernike decomposition. [11].

This study is the most extensive study examining the AUROC values of the Sirius Scheimpflug-Placido system's parameters. ROC analysis of 51 parameters was evaluated and graded them according to their AUROC value. TCT, BCV_f_, KV_f_, anterior RMS/A, BCV_b_, KV_b_, posterior RMS/A, Kmax^2^/TCT, anterior HOAs, anterior coma, and posterior coma were found to be the most valuable parameters for differentiating keratoconus eyes from normal eyes.

In the literature concerning the diagnosis of keratoconus, there is some controversy in the ROC analysis of topographic parameters. Due to variations in the settings of the topography systems and study populations, cut-off values may be different from each other in various studies.

Toprak et al. [16] reported the AUROC value of Kmax^2^/TCT as 0.997 (sensitivity >99%, specificity >94%). In our study, although the AUROC value (0.975) and sensitivity (89.6%) were not as high as those in Toprak et al. [16], the AUROC value of Kmax^2^/TCT was very close to the AUROC value of BCVf (0.976), which had the highest value in our study. We believe that Kmax^2^/TCT is a valuable parameter for discriminating keratoconus eyes from normal eyes.

RMS/A defines root mean square values per unit area in the 4.5  mm zone of the cornea and posterior RMS/A was found to be one of the most significant AUROC values (0.974) in the study. Shetty et al. reported AUROC values of 0.954 for anterior RMS/A and 0.983 for posterior RMS/A. [17] Their results are similar to ours, but their cut-off values (anterior RMS/A = 0.131 *μ*m/mm^2^ and posterior RMS/A = 0.269 *μ*m/mm^2^) are higher than our values (anterior RMS/A = 0.065 *μ*m/mm^2^ and posterior RMS/A = 0.165 *μ*m/mm^2^).

Shetty et al. [17] also reported that the AUROC values of posterior keratoconus indices (SI_b_ = 0.941; BCV_b_ = 0.969) are higher than the anterior keratoconus indices (SI_f_ = 0,921; BCV_f_ = 0.940), but we found the opposite. In our study, the AUROC values of the anterior keratoconus indices (SI_f_ = 0.950; KV_f_ = 0.974; BCV_f_ = 0.976) were higher than the AUROC values of the posterior keratoconus indices (SI_b_ = 0.936; KV_b_ = 0.965; BCV_b_ = 0.965), and BCV_f_ had the highest AUROC value.

The fact that both eyes of the patients were included in the study is one of the limitations of the study. The measurements from both eyes of the same subject tend to be positively correlated, and including only one eye per individual will give more accurate results [18]. Another limitation of the study is that keratoconus patients were not categorized in mild, moderate, and advanced cases, and the ability of the LRA model to detect subclinical keratoconus cases has not been investigated.

In conclusion, the LRA model in the study can distinguish keratoconus corneas from normal ones with high accuracy without the need for complex computer algorithms and software, but the study population is relatively small. Further studies with more patients are needed to reveal the efficacy of our results to diagnose keratoconus.

## Figures and Tables

**Table 1:** The mean values of summary indices, keratoconus indices, specially calculated indices, keratometry readings, and keratoconus indices in keratoconus and normal groups.

Parameter	Keratoconus mean ± SD (range)	Normal mean ± SD (range)	*p* value^*∗*^
*Summary indices*			
TCT (*μ*m)	445.08 ± 49.19 (212, 542)	540.28 ± 33.12 (465, 607)	0.01>
TCT *r* (mm)	0.76 ± 0.44 (0.20, 3)	0.63 ± 0.24 (0.13, 2)	0.012
CCT (*μ*m)	459.5 ± 49.95 (243, 557)	543.59 ± 31.98 (471, 611)	0.01>
*K*_max_ (D)	54.12 ± 5.71 (44.50, 68.41)	45.70 ± 2.33 (41.98, 57.07)	0.01>
Apex *r* (mm)	1.31 ± 0.79 (0.20, 3.40)	1.67 ± 0.97 (0.20, 3.40)	0.01>
CV (mm^3^)	54.03 ± 3.05 (45.6, 62.4)	58.03 ± 3.22 (49.4, 66.3)	0.01>
AD (mm)	3.3 ± 0.32 (2.57, 4.24)	3.13 ± 0.34 (2.4, 3.95)	0.01>

*Specially calculated indices*			
Kmax/TCT (D/*μ*m)	0.26 ± 1.60 (0.08, 18.1)	0.08 ± 0.007 (0.07, 0.12)	0.01>
Kmax^2^/TCT (D^2^/*μ*m)	0.049 ± 0.54 (0.0001, 6.09)	0.0001 ± 0.00002 (0.0001, 0.0002)	0.01>

*Keratometry readings*			
Sim-K1 (D)	45.01 ± 3.14 (40.11, 57.77)	42.61 ± 1.36 (39.45, 46.28)	0.01>
Sim-K2 (D)	48.19 ± 4.02 (40.92, 62.46)	43.94 ± 1.53 (41.15, 49.46)	0.01>
Anterior *Ø* = 3 mm K1 (D)	45.46 ± 5.27 (41.01, 67.90)	42.56 ± 1.35 (39.65, 46.33)	0.01>
Anterior *Ø* = 3 mm K2 (D)	49.72 ± 3.43 (40.82, 60.03)	44.06 ± 1.57 (41, 49.99)	0.01>
Anterior *Ø* = 5 mm K1 (D)	45.29 ± 4.5 (31.61, 73.12)	42.56 ± 1.33 (39.55, 46.3)	0.01>
Anterior *Ø* = 5 mm K2 (D)	49.06 ± 5.96 (40.96, 86.01)	43.95 ± 1.54 (41.06, 49.58)	0.01>
Posterior *Ø* = 3 mm K1 (D)	−6.37 ± 1.5 (−9.89, −0.26)	−5.88 ± 0.36 (−6.36, −2.57)	0.01>
Posterior *Ø* = 3 mm K2 (D)	−7.96 ± 1.69 (−15.4, −1.02)	−6.37 ± 0.39 (−9.87, −5.82)	0.01>
Posterior *Ø* = 5 mm K1 (D)	−6.26 ± 1.25 (−8.91, −0.44)	−5.9 ± 0.31 (−6.36, −3.36)	0.01>
Posterior *Ø* = 5 mm K2 (D)	−7.38 ± 1.48 (−15.88, 1.97)	−6.35 ± 0.27 (−8.06, −5.82)	0.01>

*Keratoconus indices*			
SI_f_ (D)	4.96 ± 3.68 (−7.36, 14.82)	−0.08 ± 0.56 (−1.43, 2.35)	0.01>
KV_f_ (*μ*m)	28.07 ± 17.91 (4, 102)	4.58 ± 3.65 (2, 37)	0.01>
BVC_f_ (D)	2.74 ± 1.95 (0, 11.81)	0.13 ± 0.19 (0, 1.01)	0.01>
SI_b_ (D)	1.29 ± 0.93 (−1.77, 4.46)	0.09 ± 1.37 (−0.77, 15)	0.01>
KV_b_ (*μ*m)	65.10 ± 40.18 (9, 264)	13.35 ± 9.88 (0, 111)	0.01>
BCV_b_ (D)	2.74 ± 1.90 (0, 9.83)	0.08 ± 0.15 (0, 0.80)	0.01>

^*∗*^Mann–Whitney *U* test. SD = standard deviation; TCT = minimum corneal thickness; TCT *r* = distance of the thinnest point to the center of the cornea; CCT = central corneal thickness; *K*_max_ = keratometry of the steepest point recorded from anterior tangential map; Apex *r* = distance of K_max_ to the center of the cornea; CV = corneal volume in 10 mm central corneal zone; AD = distance from the corneal endothelium to the anterior surface of the lens; Sim-K1 = simulated flat keratometry; Sim-K2 = simulated steep keratometry; K1 = flat keratometry; K2 = steep keratometry; *Ø* = central corneal zone; SI_f_ = symmetry index front; KV_f_ = keratoconus vertex front index; BCV_f_ = Baiocchi Calossi Versaci front index; SI_b_ = symmetry index back; KV_b_ = keratoconus vertex back index; BCV_b_ = Baiocchi Calossi Versaci back index.

**Table 2:** The mean values of shape indices and aberrations in keratoconus and normal groups.

Parameter	Keratoconus mean ± SD (range)	Normal mean ± SD (range)	*p* value^*∗*^
*Shape indices*			
Anterior rf (D)	46.83 ± 6.24 (30.63, 81.45)	42.59 ± 1.36 (39.63, 46.20)	0.01>
Anterior rs (D)	51.15 ± 8.60 (40.86, 113.3)	43.95 ± 1.53 (41.03, 48.91)	0.01>
Anterior Q	0.01 ± 1.17 (−5.45, 3.14)	0.84 ± 0.36 (−0.22, 1.47)	0.01>
Anterior RMS/A (*μ*m/mm^2^)	0.20 ± 0.14 (0.01, 1.17)	0.02 ± 0.02 (0.01, 0.24)	0.01>
Posterior rf (D)	−7.17 ± 1.46 (−12.76, −3.34)	−5.93 ± 0.22 (−6.38, −4.73)	0.01>
Posterior rs (D)	−7.69 ± 5.32 (−15.85, 48.20)	−6.06 ± 1.95 (−6.87, 11)	0.01>
Posterior (p)	−0.36 ± 1.60 (−3.47, 7.19)	0.80 ± 0.55 (−0.58, 4.33)	0.01>
Posterior RMS/A (*μ*m/mm^2^)	0.63 ± 0.63 (0.06, 5.69)	0.10 ± 0.06 (0.03, 0.70)	0.01>

*Aberrations*			
*Total*			
OPD (*µ*m)	2.94 ± 2.43 (0.32, 14.44)	0.95 ± 0.85 (0.25, 6.29)	0.01>
HOAs (*µ*m)	1.57 ± 1.35 (0.19, 10.18)	0.37 ± 0.42 (0.15, 4.64)	0.01>
Astigmatism Z (2, ±2) (*µ*m)	2.37 ± 2.15 (0.02, 13.37)	0.83 ± 0.78 (0.02, 4.25)	0.01>
Coma Z (3, ±1) (*µ*m)	1.10 ± 0.80 (0.08, 5.51)	0.17 ± 0.17 (0.01, 1.82)	0.01>
Spheric Z (4, 0) (*µ*m)	0.26 ± 0.34 (0.01, 2.97)	0.16 ± 0.18 (0.02, 1.74)	0.042
Residual HOAs (*µ*m)	0.89 ± 0.95 (0.13, 6.50)	0.27 ± 0.38 (0.06, 4.03)	0.01>

*Anterior*			
OPD (*µ*m)	2.84 ± 1.68 (0.37, 9.97)	0.96 ± 0.72 (0.20, 3.73)	0.01>
HOAs (*µ*m)	1.62 ± 1.09 (0.23, 8.59)	0.27 ± 0.11 (0.14, 1.05)	0.01>
Astigmatism Z (2, ±2) (*µ*m)	2.21 ± 1.49 (0.05, 9.38)	0.89 ± 0.74 (0.01, 3.71)	0.01>
Coma Z (3, ±1) (*µ*m)	1.36 ± 0.97 (0.10, 6.44)	0.15 ± 0.09 (0.02, 0.69)	0.01>
Spheric Z (4, 0) (*µ*m)	0.30 ± 0.36 (0.01, 2.87)	0.13 ± 0.04 (0.03, 0.28)	0.01>
Residual HOAs (*µ*m)	0.72 ± 0.63 (0.09, 4.96)	0.16 ± 0.10 (0.06, 0.79)	0.01>

*Posterior*			
OPD (*µ*m)	0.94 ± 0.82 (0.12, 5.71)	0.27 ± 0.40 (0.09, 4.42)	0.01>
HOAs (*µ*m)	0.75 ± 0.69 (0.08, 5.32)	0.21 ± 0.39 (0.05, 4.12)	0.01>
Astigmatism Z (2, ±2) (*µ*m)	0.49 ± 0.46 (0.01, 2.60)	0.15 ± 0.14 (0.04, 1.59)	0.01>
Coma Z (3, ±1) (*µ*)	0.35 ± 0.33 (0.02, 1.88)	0.04 ± 0.11 (0, 1.31)	0.01>
Spheric Z (4, 0) (*µ*m)	0.12 ± 0.14 (0, 1.27)	0.03 ± 0.15 (0, 1.72)	0.01>
Residual HOAs (*µ*m)	0.74 ± 1.53 (0.06, 16)	0.20 ± 0.35 (0.04, 3.64)	0.01>

^*∗*^Mann–Whitney *U* test. SD: standard deviation; rf = apical radius of the flattest meridian of the aspherotoric surface in 4.5 mm zone of the cornea; rs = apical radius of the steepest meridian of the aspherotoric surface in 4.5 mm zone of the cornea; Q = mean asphericity in 4.5 mm zone of the cornea; RMS/A: root mean square values per unit area in 4.5 mm zone of the cornea, OPD = total wavefront error; HOAs = higher-order aberrations.

**Table 3:** Receiver operating characteristic (ROC) analysis of the parameters where AUROC values are less than 0.9 95% confidence interval.

Parameter	AUROC	SE	Lower	Upper	Cut-off value	Sensitivity (%)	Specificity (%)
*Summary indices*							
TCT *r* (mm)	0.590	0.036	0.518	0.661	0.63	48.8	68.3
Apex *r* (mm)	0.616	0.037	0.544	0.688	1.50	78.4	50
AD (mm)	0.630	0.035	0.560	0.699	3.28	53.6	66.7
CV (mm^3^)	0.821	0.027	0.769	0.874	56.25	77.6	73.3

*Keratometry readings*							
Sim-K1 (D)	0.746	0.031	0.685	0.807	43.93	53.6	86.7
Sim-K2 (D)	0.873	0.022	0.829	0.917	45.32	79.2	81.7
Anterior *Ø* = 3 mm K1 (D)	0.779	0.029	0.722	0.836	43.99	56.8	88.3
Anterior *Ø* = 5 mm K1 (D)	0.748	0.031	0.687	0.810	44.05	53.6	89.2
Anterior *Ø* = 5 mm K2 (D)	0.879	0.022	0.836	0.922	45.32	80	81.7
Posterior *Ø* = 3 mm K1 (D)	0.713	0.036	0.642	0.785	−6.23	59.2	95.8
Posterior *Ø* = 5 mm K1 (D)	0.713	0.035	0.644	0.782	−6.27	54.4	96.7
Posterior *Ø* = 5 mm K2 (D)	0.886	0.023	0.840	0.932	−6.58	82.4	85.8

*Shape indices*							
Anterior rf (D)	0.802	0.029	0.745	0.858	44.04	63.2	88.3
Anterior Q	0.784	0.031	0.723	0.844	0.785	73.6	77.5
Posterior rf (D)	0.864	0.026	0.814	0.914	−6.31	71.2	98.3
Posterior Q	0.796	0.031	0.736	0.856	0.495	72.8	83.3

*Aberrations*							
*Total*							
OPD (*µ*m)	0.871	0.024	0.825	0.917	1.30	86.4	81.7
Astigmatism Z (2, ±2) (*µ*m)	0.808	0.029	0.752	0.864	1.10	76	79.2
Spheric Z (4, 0) (*µ*m)	0.575	0.039	0.498	0.652	0.205	39.2	90.8

*Anterior*							
OPD (*µ*m)	0.881	0.022	0.837	0.925	1.475	85.6	85
Astigmatism Z (2, ±2) (*µ*m)	0.809	0.028	0.755	0.864	1.285	72.8	80.8
Spheric Z (4, 0) (*µ*m)	0.638	0.038	0.563	0.713	0.215	44	96.7

*Posterior*							
Astigmatism Z (2, ±2) (*µ*m)	0.842	0.028	0787	0.898	0.245	73.6	95
Spheric Z (4, 0) (*µ*m)	0.897	0.022	0.854	0.939	0.035	75.2	93.3
Residual HOAs (*µ*m)	0.877	0.023	0.832	0.922	0.255	81.6	83.3

AUROC: area under the ROC curve; SE: standard error; Apex *r* = the distance of apex to the central cornea; AD = the depth of anterior chamber; CV = corneal volume; Sim-K1 = simulated flat keratometry; Sim-K2 = simulated steep keratometry; K1 = flat keratometry; K2 = steep keratometry; rf = apical radius of the flattest meridian of the aspherotoric surface in 4.5 mm zone of the cornea; Q = mean asphericity in 4.5 mm zone of the cornea; OPD OPD = total wavefront error; HOAs = higher-order aberrations.

**Table 4:** Receiver operating characteristic (ROC) analysis of the parameters where AUROC values are greater than 0.9 95% confidence interval.

Parameter	AUROC	SE	Lower	Upper	Cut-off value	Sensitivity (%)	Specificity (%)
*Corneal indices*							
TCT (*μ*m)	0.956	0.012	0.933	0.979	500.5	90.4	90.8
*K*_max_ (D)	0.927	0.016	0.895	0.959	47.85	84	88.3
CCT (*μ*m)	0.934	0.015	0.904	0.963	511.5	87.2	88.3

*Specially calculated indices*							
Kmax/TCT (D/*μ*m)	0.974	0.009	0.957	0.991	0.097	89.6	97.5
Kmax^2^/TCT (D^2^/*μ*m)	0, 975	0.008	0.959	0.991	0.0001	89.6	97.5

*Keratometry readings*							
Anterior *Ø* = 3 mm K2 (D)	0.901	0.020	0.862	0.940	45.60	81.6	86.7
Posterior *Ø* = 3 mm K2 (D)	0.919	0.02	0.877	0.961	−6.73	85.6	95

*Shape indices*							
Anterior rs (D)	0.909	0.019	0.871	0.947	46.36	79.2	94.2
Anterior RMS/A (*μ*m/mm^2^)	0.965	0.013	0.940	0.990	0.065	90.4	95.8
Posterior rs (D)	0.906	0.023	0.861	0.951	−6.70	84	95.8
Posterior RMS/A (*μ*m/mm^2^)	0.974	0.011	0.953	0.995	0.165	92.8	96.7

*Keratoconus indices*							
SI_f_ (D)	0.950	0.017	0.917	0.983	0.89	88.8	98.3
KV_f_ (*μ*m)	0.974	0.009	0.956	0.992	9.5	92	94.2
BCV_f_ (D)	0.976	0.01	0.956	0.996	0.51	92.8	96.7
SI_b_ (D)	0.936	0.082	0.897	0.974	0.225	88.8	98.3
KV_b_ (*μ*m)	0.965	0.013	0.939	0.990	20.5	92	95.8
BCV_b_ (D)	0.965	0.013	0.940	0.990	0.555	88.8	97.5

*Aberrations*							
*Total*							
HOAs (*µ*m)	0.940	0.016	0.909	0.972	0.60	85.6	93.3
Coma Z (3, ±1) (*µ*m)	0.933	0.017	0.899	0.966	0.39	81.6	96.7
Residual (*µ*m)	0.907	0.020	0.868	0.945	0.295	90.4	79.2

*Anterior*							
HOAs (*µ*m)	0.975	0.009	0.958	0.992	0.575	88.8	97.5
Coma Z (3, ±1) (*µ*m)	0.963	0.011	0.941	0.986	0.375	87.2	97.5
Residual (*µ*m)	0.950	0.014	0.923	0.977	0.270	89.6	89.2

*Posterior*							
OPD (*µ*m)	0.911	0.020	0.870	0.951	0.375	85.6	90
HOAs (*µ*m)	0.913	0.019	0.876	0.951	0.305	84	89.2
Coma Z (3, ±1) (*µ*m)	0.959	0.014	0.933	0.986	0.075	91.2	97.5

AUROC: area under the ROC curve; SE: standard error; TCT = minimum corneal thickness; K_max_ = keratometry of the steepest point recorded from the anterior tangential map; CCT = central corneal thickness; K2 = steep keratometry; rs = apical radius of the steepest meridian of the aspherotoric surface in 4.5 mm zone of the cornea; RMS/A: root mean square values per unit area in 4.5 mm zone of the cornea; SIf = symmetry index front; KVf = keratoconus vertex front index; BCVf = Baiocchi Calossi Versaci front index; SIb = symmetry index back; KVb = keratoconus vertex back index; BCVb = Baiocchi Calossi Versaci back index; OPD = total wavefront error; HOAs = higher-order aberrations OPD = total wavefront error; HOAs = higher-order aberrations.

**Table 5:** Performance of the logistic regression analysis models for differentiating keratoconus eyes from normal eyes.

Model	Sensitivity	Specificity	−2 log-likelihood
Model 1	%91.2	%95.8	94, 624
Model 2	%89, 6	%97, 5	98, 971
Model 3	%91, 2	%98, 3	84, 663
Model 4	%91, 2	%97, 5	79, 305
Model 5	%93, 6	% 98, 3	59, 774
Model 6	%94, 4	% 98, 3	44, 416
Model 7	%96, 8	%99.2	47, 993
Model 8	%96, 8	%99.2	42, 461

Model 1 = anterior *Ø* = 3 mm K2 + posterior *Ø* = 3 mm K2 + K_max_ + CCT + TCT; Model 2 = anterior rf + anterior rs + anterior Q + anterior RMS/A; Model 3 = posterior rf + posterior rs + posterior Q + posterior RMS/A; Model 4 = Model 2 + Model 3; Model 5 = BCV_f_ + BCV_b_ + KV_f_ + KV_b_ + SI_f_ + SI_b_; Model 6 = all of the aberrations on Table 2; Model 7 = BCV_f_ + BCV_b_ + posterior rf + KVf + anterior coma Z (3, ±1); Model 8 = BCV_f_ + BCV_b_ + posterior rf + TCT + CCT.

## Data Availability

The logistic function formula in our study can be used with an Excel file (Microsoft Office, Microsoft Corp., Redmond, WA, USA). The data used to support the findings of this study and the logistic regression formula in an Excel file are available from the corresponding author upon request.
